# Formins and membranes: anchoring cortical actin to the cell wall and beyond

**DOI:** 10.3389/fpls.2013.00436

**Published:** 2013-11-05

**Authors:** Fatima Cvrčková

**Affiliations:** Department of Experimental Plant Biology, Faculty of Science, Charles UniversityPrague, Czech Republic

**Keywords:** formin, actin, plasmalemma, endomembranes, cell polarity, endocytosis, vesicle trafficking

## Abstract

Formins are evolutionarily conserved eukaryotic proteins participating in actin and microtubule organization. Land plants have three formin clades, with only two – Class I and II – present in angiosperms. Class I formins are often transmembrane proteins, residing at the plasmalemma and anchoring the cortical cytoskeleton across the membrane to the cell wall, while Class II formins possess a PTEN-related membrane-binding domain. Lower plant Class III and non-plant formins usually contain domains predicted to bind RHO GTPases that are membrane-associated. Thus, some kind of membrane anchorage appears to be a common formin feature. Direct interactions between various non-plant formins and integral or peripheral membrane proteins have indeed been reported, with varying mechanisms and biological implications. Besides of summarizing new data on Class I and Class II formin-membrane relationships, this review surveys such “non-classical” formin-membrane interactions and examines which, if any, of them may be evolutionarily conserved and operating also in plants. FYVE, SH3 and BAR domain-containing proteins emerge as possible candidates for such conserved membrane-associated formin partners.

## INTRODUCTION

Formins (FH2 proteins) are a large family of evolutionarily conserved proteins sharing the well-defined FH2 domain (cd smart00498, pfam02181), originally identified in metazoans and fungi and later found to be ubiquitous among eukaryotes (Higgs, 2005; Higgs and Peterson, 2005; Chalkia et al., 2008; Grunt et al., 2008) and thus apparently dating back to the last eukaryotic common ancestor (see Vaškovičová et al., 2013). Land plants have three formin subfamilies, termed Class I, II and III (Deeks et al., 2002; Grunt et al., 2008), with only two of them (Class I and Class II) present in the angiosperms, and the third clade (Class III) found in mosses and lycophytes.

Formins were originally understood as multi-functional proteins involved in various aspects of cytoskeletal organization and intracellular signaling (see e.g., Frazier and Field, 1997; Heil-Chapdelaine et al., 1999). In the decade following the discovery that the FH2 domain can nucleate actin (Evangelista et al., 2002; Pruyne et al., 2002; Sagot et al., 2002) using an unique mechanism with the FH2 dimer acting as a leaky barbed end cap (Xu et al., 2004; Otomo et al., 2005), researcher’s attention shifted mainly toward their actin-related roles. However, other functions of formins are coming back into focus, in particular their participation in microtubule organization and actin-microtubule co-ordination (reviewed in Bartolini and Gundersen, 2010; Chesarone et al., 2010; Wang et al., 2012).

Another (re)emerging frequent feature of formins is their association with cellular membranes. Here studies in plants have led the way, with typical Class I formins predicted and later experimentally proven to be directly inserted into membranes, especially the plasmalemma (Banno and Chua, 2000; Cvrvčková, 2000; further experimental evidence reviewed below and in Cvrvčková, 2012 and van Gisbergen and Bezanilla, 2013). Also Class II formins often possess a domain related to metazoan phosphoinositide phosphatase PTEN, which may mediate their peripheral association with membranes (Cvrvčková et al., 2004). Indeed, in *Physcomitrella patens*, the PTEN domain of a Class II formin, For2A, was shown to bind plasmalemma phosphoinositides, especially PtdIns(3,5)P2 (van Gisbergen et al., 2012). The PTEN domain is also required for targeting the rice Class II formin FH5 to the chloroplast envelope (Zhang et al., 2011).

However, the structural and functional relationships between formins and membranes remain somewhat neglected in the literature. This review attempts to fill this gap by addressing the following questions:

(i) Which mechanisms, in addition to those described above for typical plant Class I and Class II formins, associate FH2 proteins to membranes in non-plant eukaryotic lineages?

(ii) What are the biological implications of formin-membrane association?

(iii) Which, if any, of the mechanisms and functions found in other lineages may operate also in plants?

## A VARIETY OF MECHANISMS CAN ATTACH FORMINS TO MEMBRANES

**FIGURE 1:**
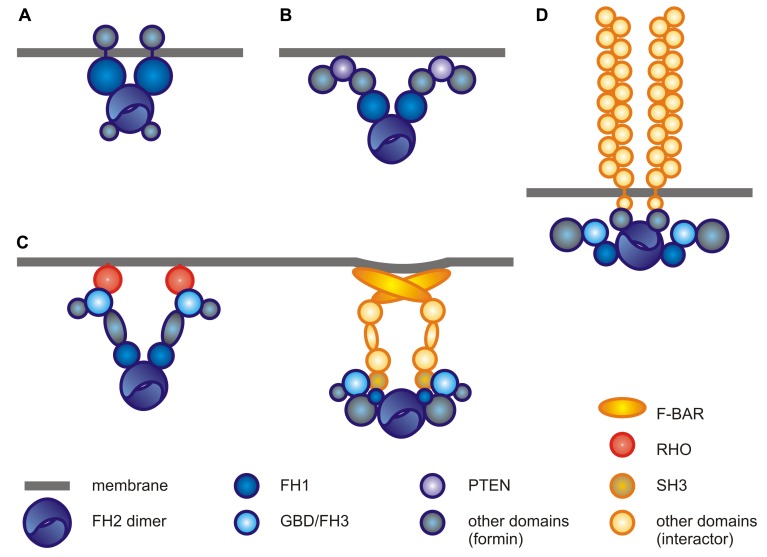
**Possible mechanisms of formin-membrane attachment.** Protein domanins are drawn roughly to scale based on the sequences of proteins listed in parentheses (including *Arabidopsis* locus identifiers and/or GenBank or Uniprot accession numbers; interacting protein pairs were chosen based on cited literature). Formins are shown in shades of blue, their interactors in shades of orange, cytoplasmic side of the membrane faces down. Complex stoichiometry is speculative in the absence of data. **(A)** Direct insertion into the membrane, as in plant Class I formins (*Arabidopsis* AtFH1, At3g25500). **(B)** Peripheral membrane binding, as in plant Class II formins (*Arabidopsis* AtFH14, At1g31810). **(C)** Interaction with a peripheral membrane protein, such as a RHO GTPase or a FBAR protein (left: mouse mDia1, NP_031884.1 and Cdc42, NP_033991.1; right: human DAAM1, XP_005267487.1, and FBP17, Q96RU3.2). **(D)** Interaction with an integral membrane protein, as in mammalian formins binding to CD21 (human FHOS, NP_037373.2, and CD21, NP_001006659.1).

The functionality (or value, in the neo-Darwinian terms) of a protein critically depends on its (intracellular) location, reminiscent of the well-known truth concerning real estate. Aside of regulating gene expression with far-reaching downstream effects, a protein can hardly exert a membrane-related function without physically associating with membranes. This may be accomplished by diverse mechanisms: by membrane insertion in integral membrane proteins, by direct binding (possibly following a post-translational modification) in peripheral membrane proteins, and, last but not least, by binding to another integral or peripheral membrane protein (**Figure 1**).

The only formins experimentally proven to be integral membrane proteins are the members of the plant Class I clade. Outside plants, secretory and transmembrane peptides were predicted only in several uncharacterized invertebrate and protist formins, without experimental proof that these proteins are membrane-located, albeit in one Caenorhabditis case there is at least cDNA evidence that the gene is expressed (Grunt et al., 2008). Some metazoan formins can also bind to membranes peripherally, similar to plant Class II formins. Drosophila Diaphanous, a prototype member of the large metazoan Diaphanous related formin (DRF) clade (Goode and Eck, 2007), directly binds PtdIns(4,5)P2 through an N-terminal basic domain. However, its membrane association requires simultaneous binding to a RHO GTPase (see below), i.e., binding a membrane phosphoinositide alone does not yet make the formin a peripheral membrane protein (Rousso et al., 2013).

Association of fungal and metazoan formins with membranes is thus usually indirect, mediated by binding to peripheral or integral membrane proteins. Numerous formin interactors have been identified, most of them cytoplasmic (Aspenström, 2010). The best characterized membrane-associated ones are notorious formin regulators – the small GTPases of the RHO family, which can attach to membranes thanks to their hydrophobic post-translational modifications. Many formins, including fungal ones and metazoan DRFs, contain a conserved N-terminal GTPase binding domain (GBD/FH3) whose binding to an active (GTP-loaded) RHO alleviates autoinhibition mediated by a C-terminal autoinhibitory domain (Watanabe et al., 1997). The GBD/FH3 domain is probably evolutionarily ancient, although it appears to be absent in plants (Rivero et al., 2005).

Formins can bind some other peripheral membrane proteins. The N-terminal portion of mammalian FMNL1, a classical GBD/FH3 containing formin, interacts with AHNAK (desmoyokin), a huge phosphoprotein binding the plasmalemma as a part of a larger multiprotein complex (Haase, 2007; Dempsey et al., 2012). Rather than attaching itself to the membrane via AHNAK, the formin, bound to a RHO GTPase, participates in recruiting AHNAK from the cytoplasm to the plasmalemma (Han et al., 2013). Association of related (FMN family) mammalian formins with compartments of the endomembrane system is, among other interactions, mediated by their binding to FYVE domain-containing proteins, including the Spir proteins that themselves can nucleate actin* in vitro* (Quinlan et al., 2005; Kerkhoff, 2011; Dietrich et al., 2013).

Proteins containing the conserved F-BAR domain, an extended version of the membrane-binding BAR domain (Heath and Insall, 2008; Roberts-Galbraith and Gould, 2010) may also contribute to interaction-mediated membrane localization of formins (albeit also here the localization may work in both directions, as F-BAR proteins are involved in large multiprotein complexes including RHO GTPases as well). Yeast and mammalian formin interactors such as FBP1/FBP17/Rapostlin (Wakita et al., 2011), FNBP1L/Toca (Huett et al., 2009) or CIP4 (Aspenström et al., 2006) all share a common architecture with an N-terminal F-BAR domain and C-terminal SH3 domain, with a coiled coil motif in between (Roberts-Galbraith and Gould, 2010). A mammalian homolog of CIP4, a prototype protein of this family originally identified as a Cdc42 (RHO GTPase) effector, interacts with the DAAM1 formin via its SH3 domain, raising thus the possibility that other SH3-containing proteins may bind formins as well (Aspenström et al., 2006). This is not surprising, as SH3 domains associate with proline-rich proteins (Alexandropoulos et al., 1995), and the majority of formins contain an extremely Pro-rich domain, termed FH1, in front of the hallmark FH2 domain. Indeed, the same study identified a Src family non-receptor tyrosine kinase as a DAAM1 binding partner, confirming thereby previous observations that other metazoan formins can bind Src (Uetz et al., 1996).

SH3 domain-containing proteins often interact with integral membrane proteins, and some are themselves inserted into membranes, such as, e.g., the budding yeast protein Fus1p (not to be confused with the fission yeast formin Fus1) which can bind to the Bni1p and Bnr1p formins via its SH3 domain (Tong et al., 2002). Another SH3-containing transmembrane protein, the osmosensor Sho1p, participates in a larger protein complex with Bni1p and Fus1p (Nelson et al., 2004).

Additional integral membrane proteins directly bind formins. The zebrafish plasmalemma protein Antxr2 (anthrax toxin receptor 2) participates in a ternary complex involving also a RHO GTPase and a DRF type formin (Castanon et al., 2013). The glutamate receptor delta2 (Grid2) from mammalian neurons binds to delphillin, an unusual formin that contains a PDZ domain that appears to be required for this apparently delphillin-specific interaction (Miyagi et al., 2002). In *Aspergillus*, MesA, a protein possibly post-translationally inserted into membranes, may be contributing to the localization of the SepA formin in the plane of the membrane (Pearson et al., 2004). Even a membrane transporter – the PKD2 cation channel – was reported to bind a DRF type formin (Rundle et al., 2004). Remarkably, the cytoplasmic domain of human complement receptor 2 (CD21) binds to the FHOS/FHOD1 formin and facilitates its localization to the plasmalemma upon viral infection (Gill et al., 2004), demonstrating that interactions with membrane proteins can indeed recruit formins to membranes.

Formins in non-plant lineages thus appear to have explored in evolution a variety of membrane association mechanisms which have not been documented, or even suspected, to exist in plants.

## WHAT ARE THEY DOING THERE: NON-PLANT FORMINS IN MEMBRANE TRAFFICKING

Detailed discussion of the RHO-controlled, actin nucleation or actin-microtubule co-ordination-based cortical processes in non-plant lineages, including formation of invasive structures such as e.g., metazoan filopodia, would be out of scope of this review, and can be found elsewhere (e.g., Chesarone et al., 2010; Yang and Svitkina, 2011; Vaškovičová et al., 2013). What follows is a summary of biological implications of the formin-membrane interactions discussed in the previous section.

Some of these mechanisms may localize formins within the plane of the plasmalemma, participating thus in the control of cell polarity, or delimiting cell surface domains with increased membrane expansion or turnover (including polar or tip growth; for the concept of “activated cortical domains” in plant cells compare Žárský et al., 2009). Phosphoinositide interaction of Drosophila Diaphanous is required for targeting the formin to the epithelial apical membrane (Rousso et al., 2013), and interaction with the F-BAR protein CIP4 may inhibit Diaphanous in lateral and basal membrane domains (Yan et al., 2013). However, other metazoan F-BAR proteins may stimulate formin activity while connecting the plasmalemma and the cortical cytoskeleton during actin-driven membrane tubulation and ruffling (Toguchi et al., 2010) or during formation of dendritic spines in neurons (Wakita et al., 2011). *Aspergillus* formin interactor MesA promotes formin localization to growing tips of hyphae (Pearson et al., 2004), reminiscent of the function of some plant formins in tip growth (see below). Similarly, formin-containing complexes of budding yeast Fus1p localize at the tip of mating protrusions, or “shmoos” (Nelson et al., 2004). In zebrafish, complexes involving RHO, a DRF type formin and Antxr2a exhibit polar localization at the plasmalemma and contribute to division plane positioning (Castanon et al., 2013).

Formins also associate with the endomembrane system and participate in vesicle trafficking. The above-described metazoan Spir/formin complexes engage in actin-dependent vesicle transport, possibly via actin nucleation on vesicle membranes (see Kerkhoff, 2011; Dietrich et al., 2013). Formins, bound to RHO GTPases, also participate in spatially restricted endocytosis and in endosome dynamics in both yeasts (Gachet and Hyams, 2005; Prosser et al., 2011) and metazoans, where interaction with Src appears to be contributing as well (Gasman et al., 2003). It has to be noted, though, that all the endosome- and endocytosis-associated formins described so far contain the GBD/FH3 domain which can engage in endocytosis regulation also outside the formin context, as in the Entamoeba EhNCABP166, which lacks the FH2 domain (Campos-Parra et al., 2010). The F-BAR family formin interactors are also predominantly involved in endocytosis (Feng et al., 2010), as well as in autophagy, also an endosome-dependent process (Huett et al., 2009). The F-BAR domain’s ability to increase or stabilize membrane curvature may play an important role in generating endocytotic membrane vesicles, a process facilitated by dynamin (Roberts-Galbraith and Gould, 2010).

While most reports on formin-endomembrane associations point to endocytotic pathways or compartments, genetic data from fission yeast suggest that the For3 formin participates in exocytosis, as a synthetic thermosensitivity phenotype was observed upon combining mutations affecting For3 and Mug33, a transmembrane protein involved in polarized secretion and co-localizing with the exocyst complex (Snaith et al., 2011). Also the formin binding partner AHNAK has been implicated in the delivery of Ca2+ channels to the plasmalemma repair of cell membrane lesions, i.e., in processes that, on the first glance, appear to be exocytosis-driven, albeit they have a non-separable endocytotic component as well (Idone et al., 2008).

To summarize, numerous lines of evidence point to formins being involved in various aspects of endosome trafficking or endomembrane system organization. Recent reports even indicate that the ER associated formin INF2 (Chhabra et al., 2009) participates in the division of mitochondria, which involves a dynamin-related protein (Korobova et al., 2013), and other formins contribute to actin rearrangements involved in Toxoplasma apicoplast division (Jacot et al., 2013). However, as most of the reported interactions involve proteins so far found only in opisthokonts, it remains to be seen if similar mechanisms operate also in plants.

## MEMBRANE-ASSOCIATED FORMINS IN PLANTS: THE KNOWN AND THE POSSIBLE

Insertion of typical plant Class I formins into membranes, as well as membrane association of PTEN domain-containing formins, is experimentally well documented. As far as biological function is concerned, plant formins, often plasmalemma-associated, were shown to participate in the control of the cell cortex architecture during cell growth, including both tip growth (e.g., Cheung and Wu, 2004; Deeks et al., 2005; Yi et al., 2005; Vidali et al., 2009; Ye et al., 2009; Cheung et al., 2010) and isodiametric or polar expansion (Favery et al., 2004; Rosero et al., 2013), as well as in cytokinesis (Ingouff et al., 2005; Li et al., 2010). The *Arabidopsis* AtFH1 formin mediates trans-membrane anchorage of the cortical actin to the cell wall, exhibits restricted lateral mobility due to its cell wall attachment, and localizes to microtubule-free cortical areas (Martiniere et al., 2011, 2012), providing thus a possible mechanism for attenuating cortical microtubule dynamics. Consistent with this hypothesis, mutants lacking AtFH1 have more dynamic microtubules (Rosero et al., 2013).

Similar to other eukaryotic lineages, also in plants formins may be closely involved in membrane turnover or associated with endomembranes. *Physcomitrella patens* Class II formin For2A specifically localizes to PtdIns(3,5)P2-rich sites of active plasmalemma turnover (van Gisbergen et al., 2012). Overexpressed microtubule-associated Class I *Arabidopsis* formin AtFH4 can decorate the endoplasmic reticulum and co-align it to the microtubule cytoskeleton (Deeks et al., 2010), and its relative AtFH8 is targeted to the nuclear envelope (Xue et al., 2011). Loss of tip polarity in formin-overexpressing pollen tubes (Cheung and Wu, 2004; Cheung et al., 2010) or root hairs (Yi et al., 2005), as well as irregular cell wall thickening observed in rice mutants lacking the Class II formin FH5 (Yang et al., 2011) might be understood as disturbance of the exocytosis/endocytosis co-ordination. Thus, the biological implications of formin-membrane association may be conserved, and it is worth examining the molecular mechanisms underlying membrane localization of formins.

Non-classic angiosperm formins lacking the transmembrane (in Class I) or PTEN-like (in Class II) domains might heterodimerize with their membrane-bound paralogs. Surprisingly, FH2-mediated formin heterodimerization has been neither documented nor excluded yet in any organism, albeit dimerization via other domains was reported (see Cvrvčková, 2012).

The Rop GTPases represent a plant branch of RHO proteins (see Mucha et al., 2011), often understood as general formin regulators. However, plant formins lack the RHO-binding GBD/FH3 domain, and the only putative RHO interaction motif found in land plant FH2 proteins is a RHO GTPase activating protein (RhoGAP)-related domain in non-angiosperm Class III formins (Grunt et al., 2008). Thus, Rops are unlikely to provide the means for direct formin-membrane binding in angiosperms, albeit they may participate in larger multi-subunit complexes.

Few, if any, clear homologs of other non-plant membrane associated formin interactors can be identified in database searches (**Table 1**). Two protein families may, nevertheless, deserve a closer look.

**Table 1:** Candidate plant membrane-associated formin interactors.

Protein or domain(s)	Non-plant query	Land plant candidates	Notes
AHNAK	NP_001611.1 (human AHNAK isoform 1)	N.A.	Best plant BLAST hit with E-value 5e-06 only matches a low compexity region of AHNAK
Spir (FYVE)	NP_001246101.1 (Drosophila spire isoform F)	N.A.	
other FYVE	cd00065 (FYVE domain)	At4g33240, FAB1A; At3g14270, FAB1B	Many plant FYVE domain protein exist; for candidate selection see text.
F-BAR-SH3	NP_004231.1 (human CIP4); NP_055848.1 (human FBP1); NP_060207.2 (human FNBP1)	N.A.	No* bona fide* plant F-BAR domains but several proteins have an analogous BAR-SH4 domain layout with a plant-specific shorter BAR domain (cd07607) instead of FBAR (see BAR-SH3).
Fus1 (SH3)	NP_009903 (*Saccharomyces cerevisiae* Fus1p)	N.A.	
other BAR-SH3	cd07607 (BAR domain of the plant SH3 domain-containing proteins)	At1g31440, AtSH3P1 At4g34660, AtSH3P2 At4g18060, AtSH3P3	No additional *Arabidopsis* paralogs identified by Blast with AtSH3P3 query.
Antxr2	XP_005165376.1 (zebrafish Antxr2a isoform X1)	N.A.	
MesA	Q5BGR2.2 (*Aspergillus nidulans* MesA)	N.A.	
Grid2	NP_001501.2 (human Grid2)	Numerous glutamate receptors exist in plants but formin association unlikely.	PDZ domain in the formin partner required for binding, not founds in plant formins.
CD21	NP_001006659.1 (human CD21 isoform 1)	N.A.	
PKD2	NP_032887.3; (mouse polycystin-2)	N.A.	PKD2 homologs found in Micromonas and volvocal algae.

*GenBank/Uniprot accession numbers are provided for protein sequences used as queries, and NCBI conserved domain database accessions for domains. N.A., not available (not found in standard Blast searches of the Viridiplantae section of the NCBI protein database using the listed non-plant sequences as queries). For proteins and domains where land plant candidates were found, only Arabidopsis proteins are shown (referred to using standard A. thaliana locus nomenclature), albeit non-Arabidopsis homologs without experimental data exist as well.*

While there is no direct plant homolog of Spir, numerous plant proteins harbor FYVE domains. The 15 FYVE-containing proteins of *A. thaliana* can be divided into five groups according to their domain architecture (Wyvial and Singh, 2010). Most of these proteins are experimentally uncharacterized, and none exhibit a significant match to any of the previously described formin interactors in BLAST searches. However, the only two experimentally characterized *Arabidopsis* FYVE-containing proteins encoded by the FAB1A and FAB1B genes are members of type III phosphatidylinositol 3-phosphate 5-kinase, or PIKfyve, family which has been implicated in endocytosis and actin dynamics in metazoan cells, albeit with no evidence for direct formin participation (Shisheva, 2008). Intriguingly, in *Arabidopsis*, mutations in FAB1A/B cause extensive vacuolization and collapse of pollen grains (Whitley et al., 2009), disrupt endocytosis and vacuole pH regulation, and perturb auxin transporter recycling (Hirano and Sato, 2011; Hirano et al., 2011; Bak et al., 2013). While these effect may be due to various regulatory effects of PtdIns(3.5)P2 produced by PIKfyve, a possible involvement of formins (including Class II members binding to PtdIns(3.5)P2-containing membranes) may deserve attention.

Likewise, no direct homolog of yeast Fus1p (a transmembrane SH3-containing protein) has been found. However, members of the coiled-coil-SH3-containing family of AtSH3Ps associate with the plasmalemma and endomembranes and participate in clathrin-mediated endocytosis (Lam et al., 2001), albeit there is yet no evidence of their interaction with formins. AtSH3P2 appears to be upregulated in pollen tubes, whose growth is formin-dependent (Wang et al., 2008). Intriguingly, these proteins contain a N-terminal BAR domain, a plant-specific variant of a shorter version of the F-BAR domain (which is absent in plants); and perhaps they might represent a plant counterpart of the yeast and metazoan F-BAR formin interactors.

Last but not least, plant formins may be attached to membranes by lineage-specific mechanisms. A gene encoding a protein with unique combination of FH2 and Sec10 domains, physically linking a formin and a subunit of the membrane-associated Exocyst complex, exists in Physcomitrella (Grunt et al., 2008; Cvrvčková, 2012), and the first identified plant formin interactor, FIP2 (At5g55000; Banno and Chua, 2000) contains a domain corresponding to the oligomerization interface of voltage-gated potassium channels, and might perhaps interact with them.

In summary, there may be more to the association of plant formins with membranes than just the transmembrane and PTEN-like domains characterizing the two angiosperm formin clades, and a comparison with non-plant systems does provide some candidates that may be worth closer investigation.

## Conflict of Interest Statement

The authors declare that the research was conducted in the absence of any commercial or financial relationships that could be construed as a potential conflict of interest.
